# Aberrant Activation of NF-κB Signalling in Aggressive Lymphoid Malignancies

**DOI:** 10.3390/cells7110189

**Published:** 2018-10-30

**Authors:** Ruth Kennedy, Ulf Klein

**Affiliations:** Experimental Haematology, Leeds Institute of Medical Research at St. James’s, School of Medicine, University of Leeds, Leeds LS9 7TF, UK; r.m.kennedy@leeds.ac.uk

**Keywords:** nuclear factor-κB, B-cell lymphoma, germinal center, lymphomagenesis, therapeutic

## Abstract

Lymphoid malignancies frequently harbor genetic mutations leading to aberrant activation of nuclear factor-κB (NF-κB) signaling; in normal cells, this pathway has important roles in the control of cell growth, survival, stress responses, and inflammation. Malignancies with mutations in NF-κB pathway components can derive from all cell stages of mature B-cell development; however, aberrant NF-κB activity is particularly prevalent in aggressive subtypes of non-Hodgkin lymphoma and myeloma. NF-κB activation is mediated by two separate pathways, the canonical and alternative pathway, and five downstream transcription factor subunits. Recent findings implicate a predominant role for distinct NF-κB pathways and subunits in certain lymphoma subtypes and myeloma; findings which are complemented by the realization that individual NF-κB subunits can have unique, non-redundant biological roles in the putative tumor precursor cells, including activated B cells, germinal center B cells and plasma cells. The knowledge gained from these studies may be exploited for the development of therapeutic strategies to inhibit aberrant NF-κB activity at the level of the transcription-factor subunits and their target genes, as global inhibition of the pathway is toxic. Here, we provide an overview on the role of aberrant NF-κB activation in aggressive lymphoid malignancies and discuss the potential importance of individual NF-κB subunits in the pathogenesis of tumor subtypes.

## 1. Introduction

Cancers of the B-cell lineage arise from malignant transformation of B cells and plasma cells (PCs) at various stages of differentiation. The majority of B-cell lymphomas and PC malignancies originate from B cells that have undergone the germinal center (GC) B-cell reaction of the T cell-dependent immune response that is essential for our immunity against foreign antigens [1,2]. During the GC reaction, B cells recognizing the invading pathogen undergo somatic hypermutation in their rearranged immunoglobulin variable (IgV) genes in order to generate high-affinity PCs and memory B cells [3,4]. Another process occurring during the GC reaction is Ig class switch recombination, in which the B cell switches from the expression of IgM to other Ig classes with different effector functions. Errors during the DNA-modifying processes of somatic hypermutation and class switch recombination, in addition to mistakes occurring during the rearrangement of the antibody genes in developing B cells, can cause genetic aberrations (e.g., chromosomal translocations and deletions) that activate oncogenes or inactivate tumor suppressor genes [5]. A common pathogenic mechanism in the genesis of lymphomas and PC malignancies is the deregulation of proto-oncogenes (e.g., *MYC*, *BCL2*, *BCL6*) and the inactivation of tumor suppressor genes (e.g., *PRDM1*) that have crucial functions in the control of normal B-cell and PC development through genetic aberrations [6]. Recently, deregulated activity of the NF-κB signaling pathway due to genetic mutations emerged as a major driver in the pathogenesis of several B-lineage malignancies, especially in aggressive subtypes of B-cell non-Hodgkin lymphoma (B-NHL) and in multiple myeloma (MM), an incurable tumor of PCs.

## 2. NF-κB

**Figure 1:**
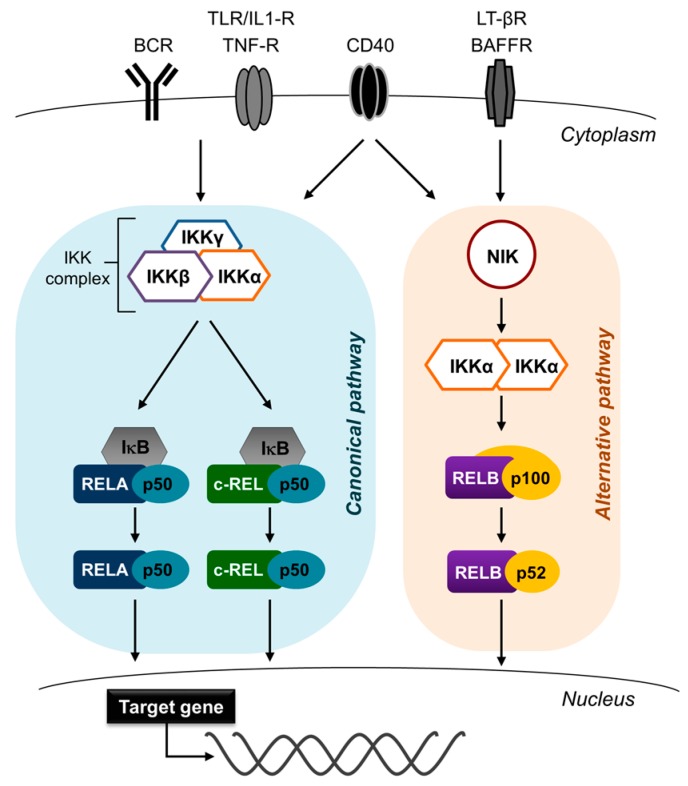
Nuclear factor-κB (NF-κB) signaling pathway. The NF-κB signaling cascade comprises two separate pathways. The canonical pathway (**left**) is activated by signals through several cell surface receptors, including the B-cell receptor (BCR), Toll-like receptor, and CD40. The activation results in the nuclear translocation of the heterodimers constituted by the canonical NF-κB subunits RELA, c-REL and p50 where they activate the transcription of target genes. The alternative pathway (**right**) is activated by B cell-activating receptor (BAFFR), lymphotoxin-β receptor (LT-βR), and CD40. Proteasomal degradation of p100 results in the generation of the major heterodimer of the alternative pathway RELB/p52, which translocates to the nucleus and activates target-gene transcription. Only RELA, RELB, and c-REL can transcribe target genes due to transactivation domains.

The NF-κB signaling pathway plays a crucial role in multiple biological processes, including survival, growth, inflammation and stress responses, across many cell types, through regulation of gene expression [7,8]. NF-κB signaling ultimately leads to the activation of five NF-κB transcription factor subunits, c-REL, RELA (p65), RELB, p50 (and its precursor p105), and p52 (and its precursor p100) which associate as hetero or homodimers (Figure 1). The c-REL, RELA and RELB subunits are the drivers of transcription through ‘transactivation domains’ at the C-termini. Receptor-mediated activation of the dimers causes their translocation to the nucleus, where they regulate the expression of NF-κB target genes. The NF-κB transcription factors cluster into two groups; the RELA, c-REL and p50 subunits mediate the canonical NF-κB pathway, which in B cells is activated by stimulation through the B-cell receptor (BCR), Toll-like receptors and CD40 ligation [9,10]. The major heterodimers are RELA/p50 and c-REL/p50; these are retained in the cytoplasm by inhibitory κBα (IκBα) and released following IκB kinase (IKK)-mediated phosphorylation and subsequent proteasomal degradation of IκBα, causing the translocation of the heterodimers to the nucleus. Nuclear translocation of NF-κB transcription factors thus indicates NF-κB pathway activation. The RELB/p52 heterodimer of the alternative (or non-canonical) pathway is activated in B cells by CD40 ligation, B-cell activating factor (BAFF) or lymphotoxin-β (LTβ) receptor [9,10,11]. Activation of the alternative pathway results in the release and stabilization of NF-κB-inducing kinase (NIK) from the TRAF2/TRAF3/cIAP1/2 complex [12]. NIK activates IKKα, which in turn induces cleavage of p100 (which is bound to RELB) by phosphorylation; this allows for nuclear translocation of the RELB/p52 heterodimer. In certain cell systems, crosstalk between the pathways may occur. Thus, NIK has been found to also activate the canonical pathway [13,14,15,16], and in some contexts, canonical NF-κB subunits can transcriptionally upregulate expression of the alternative subunits [17]. Additionally, CD40 is known to activate both the canonical and alternative NF-κB pathways (Figure 1) [9,10]. A potential crosstalk between the two pathways may need to be taken into consideration when interpreting the downstream effects of a particular NF-κB-inducing stimulus.

## 3. Germinal Center Reaction and NF-κB

While the roles of NF-κB in early B-cell development and in the survival of naïve B cells (the precursors of GC B cells) have been firmly established [9,10,18], the function of NF-κB in the later stages of mature B-cell development—encompassing the GC reaction and differentiation of GC B cells into memory B cells or PCs—could not be investigated for technical reasons, in particular since constitutional knockout mice of NF-κB subunits either lack or have severely impaired GC formation [9,10,18]. Recent studies could provide new insights into NF-κB activation and the roles of the separate NF-κB transcription factor subunits during late B-cell differentiation, which comprises the cellular counterparts of the various types of lymphomas and PC malignancies.

The GC, which is the place where the adaptive immunity in the antibody system is generated [19], is a transient structure developing within peripheral lymphoid organs in response to invading pathogens [3]. Guided by T cell help, antigen-activated B cells migrate from the interfollicular region to the primary follicle. The B cells rapidly divide within the follicle until day 7 of the GC response, when a mature, polarized GC microenvironment is formed, which comprises two functionally distinct compartments—a dark zone and a light zone [4,20,21,22]. Dark zone GC B cells proliferate vigorously and somatically hypermutate their IgV genes, thus generating a large number of antibody mutants in a short time frame. These cells move to the light zone where B cells are selected for improved antibody affinity, a process that involves follicular dendritic cells and T follicular helper (Tfh) cells. Many of these B cells undergo apoptosis as the IgV gene mutations negatively impact antigen-binding or the structure of the antibody. Positively selected B cells either recirculate back to the dark zone to undergo further rounds of proliferation and IgV hypermutation [4,20,21,22], or differentiate into PCs or memory B cells [23,24,25]. A large fraction of GC B cells also undergo Ig class switch recombination. The initiation and maintenance of the GC reaction and post-GC differentiation are the subject of transcriptional, post-transcriptional and epigenetic control [21]. The transcriptional control of the GC reaction is increasingly well understood. The transcriptional repressor B-cell lymphoma 6 (BCL6) is considered the master regulator of the GC reaction, since it is essential for the establishment of the unique dark zone program that allows for rapid proliferation and the introduction of somatic hypermutations into the IgV genes without eliciting a DNA damage response [26]. B lymphocyte-induced maturation protein 1 (BLIMP1; encoded by *PRDM1*) and interferon regulatory factor 4 (IRF4) are important transcription factors at the GC exit; X box-binding protein 1 (XBP1) is downstream of IRF4 and BLIMP1 and sets up the secretory program of the PC [23]. c-MYC, which is expressed in a small fraction of light zone B cells, is required for the recirculation of antigen-selected B cells back to the dark zone [27,28]. Abolishing the function of c-MYC in light zone B cells leads to the collapse of the GC structure, pointing towards the essential role of this B-cell subset for the maintenance of the GC reaction. With regards to NF-κB, studies in humans have provided insights into the expression pattern of NF-κB in the GC.

**Figure 2:**
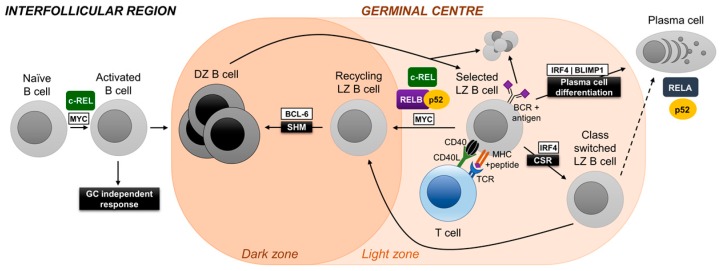
Role of NF-κB subunits in the molecular control of germinal center (GC) initiation, maintenance and differentiation. High-affinity PCs and memory B cells are generated in the GC B-cell reaction (see text). At day 7 of the GC response, a mature, polarized GC microenvironment is formed which comprises of a dark zone and a light zone. Dark zone GC B cells somatically hypermutate their immunoglobulin variable (IgV) genes and move to the light zone where B cells are selected for improved antibody affinity, a process that involves follicular dendritic cells and T follicular helper (Tfh) cells. Positively selected B cells either recirculate back to the dark zone to undergo further rounds of proliferation and IgV hypermutation, or differentiate into PCs and memory B cells. c-REL is required for B-cell activation, whereas RELA is dispensable at this stage. c-REL, and independently RELB/p52, are required for the recirculation of an antigen-selected GC B cell from light to dark zone, and thus for the maintenance of the GC reaction. Conversely, RELA is required for the differentiation of a GC B cell into a PC precursor (plasmablast). The p52 subunit alone (i.e., it does not require RELB) is essential for the physiology of PCs. Figure adapted from [21].

In humans, the vast majority of GC B cells are not subjected to NF-κB signaling; this includes dark zone and most light zone B cells [29,30]. However, similar to c-MYC, NF-κB activation was observed in a small subpopulation of light zone B cells, presumably via stimulation of the BCR and the CD40 signal-transduction pathway, resulting in the nuclear translocation of canonical and alternative NF-κB subunits [30,31]. This selective activation of NF-κB in a subset of light zone B cells suggests roles for NF-κB transcription factor subunits in GC biology, and was experimentally addressed through the specific in vivo ablation of the individual NF-κB subunits using conditional alleles and a Cre-recombinase that is expressed in GC B cells [31,32,33]. These studies, perhaps surprisingly, have revealed that the separate NF-κB transcription factor subunits exert distinct biological functions during GC and post-GC B cell development (Figure 2).

### 3.1. c-REL

*Rel* (c-REL) constitutional knockout mice generate a naïve B-cell repertoire comparable to their wild-type counterparts [34,35]. However, in vitro mitogen-stimulation experiments revealed the requirement of c-REL during B-cell activation. Accordingly, *Rel* knockout mice showed impaired formation of GCs following T-dependent immunization [36]. This is intrinsic to B cells, since GC formation was strongly impaired in conditional *Rel* knockout mice with deletion of *Rel* in all B cells using a CD19-Cre allele [37]. The role of c-REL during the GC reaction was investigated through the use of conditional *Rel* knockout mice that expressed the Cre-recombinase in GC B cells (Cγ1-Cre mice) [32]. c-REL ablation in GC B cells led to the gradual collapse of the GC after day 7, which is the time-point at which dark and light zones have formed and selection is thought to begin. Loss of dark zone and light zone cells in c-REL-deficient GCs was concurrent and led to the almost complete disappearance of GCs in the *Rel* conditional mice at day 14. These findings are reminiscent of those of the GC-specific ablation of c-MYC [27,28] and suggest that also c-REL is required for cyclic re-entry of antigen-selected B cells from the light zone to the dark zone. Gene expression profiling of c-REL-deficient GC B cells suggests that c-REL is required in light zone B cells to establish a metabolic program that generates energy and building blocks to facilitate cell growth [32]. In agreement with these observations, in vitro-stimulated c-REL-deficient B cells were characterized by reduced metabolic activity compared to wild-type B cells. While it is unclear to what extent c-MYC and c-REL crosstalk among each other, an NF-κB signature is present in the c-Myc^+^ light zone subset [28], suggesting that c-REL and c-MYC are active in the same subset of cells. A recent study that provides evidence that GC B cells rewire their BCR and CD40 signaling to enhance selection stringency in the GC suggests that the CD40-mediated activation of NF-κB by Tfh cells is jointly required with BCR activation (which, unlike in naïve B cells, does not activate NF-κB in GC B cells) to induce c-MYC expression in GC B cells [38]. In summary, c-REL shows a biphasic activation pattern at two stages of the GC reaction, as it is required during the T cell-dependent antigen-activation phase preceding GC formation, and then several days later in the fully established GC during the selection of light zone B cells for high-affinity antibodies.

### 3.2. NF-κB1

The inhibition of IKK complex-induced proteolysis of p105, which is the precursor of p50, was found to impair the antigen-induced formation of GCs in murine B cells, similar to what has been observed for *Rel* deletion in B cells [39]. Thus, the phenotype in the p105 mutant mice may be due to their inability to process p105, which in turn prevents the formation and ultimately the nuclear translocation of c-REL/p50 heterodimers. Conversely, the loss of p105 (which essentially is an inhibitory κB protein for c-REL and RELA) in *Nfkb1*^–/^^–^ mice (*Nfkb1* is the gene encoding p105/p50) may lead to enhanced c-REL activity in B cells, which might contribute to the increased formation of spontaneous GCs that has been observed in aging NF-κB1-deficient mice [40].

### 3.3. RELA

Germline deletion of *Rela* (RELA) results in embryonic lethality at day 15 [41]. Experiments with irradiated SCID mice reconstituted with *Rela*^–^ fetal liver cells suggested that c-REL and RELA are redundant during the generation of the naïve B-cell repertoire [42], a finding that was confirmed in conditional *Rel* and *Rela* knockout mice crossed to CD19-Cre mice [37]. However, in contrast to c-REL, RELA was dispensable for both the formation of GCs [37] and, as investigated by crossing the conditional allele to Cγ1-Cre mice, for GC maintenance [32]. Intriguingly, the GC B cell-specific deletion of *Rela* abolished the generation of GC-derived PCs [32]. This may at least in part be due to a role of RELA in upregulating the expression of the PC master regulator BLIMP1 [32]. Of note, mRNA and protein expression of RELA’s canonical counterpart c-REL is strongly downregulated in normal human and murine PCs [31,43], indicating that RELA is the exclusive transcriptionally active canonical NF-κB subunit in PCs.

### 3.4. RELB and NF-κB2

Combined GC-specific deletion of *Relb* (RELB) and *Nfkb2* (p100/p52), but not that of the single alternative NF-κB subunits, resulted in the collapse of established GCs [31], similar to what has been observed for *Rel* deletion [32]. This suggests that the alternative NF-κB pathway is required for GC maintenance in a non-redundant fashion with c-REL. RELB/p52 was found to be required for cell-cycle progression. Moreover, evidence suggests that RELB/p52 is required in differentiating GC B cells to set up a program that allows for the efficient production of proteins and facilitates antibody secretion. Of note, p100/p52 was found to be strongly expressed in both PC precursors in the GC and in subepithelial PCs in human tonsil compared to surrounding lymphocytes [31]. In agreement with this finding, the deletion of *Nfkb2*, but interestingly not that of *Relb*, in GC B cells led to a dramatic reduction in antigen-specific PCs [31], suggesting a critical role for p52 in the development and/or physiology of PCs.

## 4. Aberrant Activation of NF-κB in Lymphoid Malignancies

The development of lymphoid malignancies is mechanistically tied to the deregulation of cellular pathways, which govern the differentiation, proliferation, and survival of lymphocytes or PCs [6,44,45]. The NF-κB signaling pathway has functions in all of these cellular pathways. Perhaps not surprisingly, constitutive NF-κB activity occurs in many lymphoid malignancies and can be activated by the tumor microenvironment, tumor viruses or as the results of genetic alterations in NF-κB pathway components. Especially the latter has become increasingly well understood over the last decade primarily, due to next-generation sequencing efforts that could provide a clear pattern of the genetic aberrations leading to aberrant NF-κB activity in the various lymphoid malignancies. From these studies, it has emerged that NF-κB’s role in lymphomagenesis varies depending on the tumor subtype; an overview of subtypes, characteristics, genetic aberrations and NF-κB pathway involvement is presented in Table 1.

**Figure 3:**
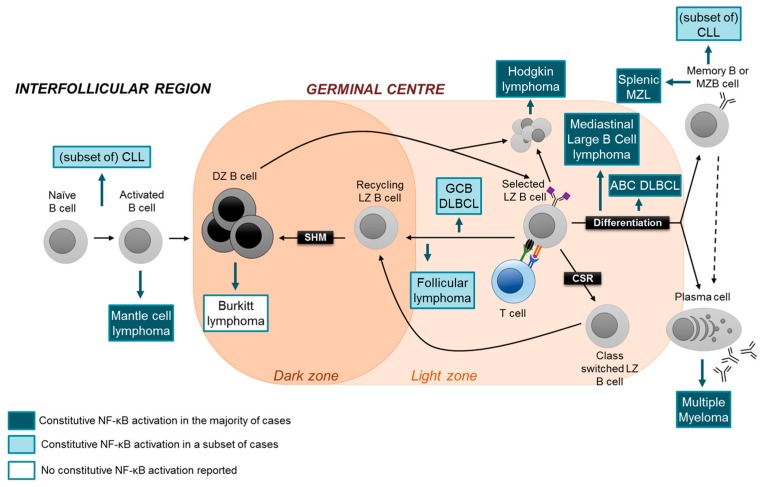
GC B-cell development and cellular derivation of B-lineage malignancies. Whereas Burkitt lymphoma seems to originate from the transformation of dark zone cells, recent evidence suggests that follicular lymphoma and germinal center B-cell type (GCB)-DLBCL may derive from the transformation of light zone cells (see text). Activated B-cell type (ABC)-DLBCL is thought to originate from light zone cells poised to undergo PC differentiation. Conventional mantle cell lymphoma (MCL) may derive from the oncogenic transformation of an antigen-activated pre-GC B cells. Multiple myeloma is a tumor of *bona fide* PCs. Hodgkin lymphoma is thought to originate from light zone B cells with “crippled” antigen receptors, mediastinal large B-cell lymphoma from a differentiation light zone B cell, and MZL from a post-GC marginal zone/memory B cell. Chronic lymphocytic leukemia (CLL) can have unmutated and mutated IgV genes. Several mature B-lineage malignancies show constitutive NF-κB activity, as indicated.

It has long been established that lymphomas and PC malignancies originate from the malignant transformation of B cells and PCs at different stages of cellular differentiation (Figure 3). The heterogeneity of lymphoma subtypes appears to be reflected by the marked heterogeneity of GC B-cell subtypes. For example, whereas Burkitt lymphoma is consider to be a tumor of dark zone B cells, follicular lymphoma and the GC-type of diffuse large B-cell lymphoma (GCB-DLBCL) are thought to originate from the transformation of light zone B cells, and the normal counterpart of the activated B cell-type DLBCL (ABC-DLBCL) may represent a differentiated late GC B cell destined to become a PC [44]. Extrapolating the findings that canonical and alternative NF-κB subunits have distinct functions in the various B-cell subsets which represent the normal counterparts of lymphoid malignancies, identifying the roles of the individual oncogenic NF-κB transcription factors in these tumor subtypes may be exploited for the development of more specific therapies that target aberrant NF-κB activity. In the following, we will discuss the role of aberrant NF-κB activation in lymphomas and PC malignancies with a particular emphasis on the potential involvement of the separate NF-κB pathways and subunits in the disease process. Thereby, a special focus will be on MM and subtypes of aggressive B-NHL, where oncogenic NF-κB signaling due to genetic mutations is particularly prevalent. For a detailed discussion of the role of NF-κB in various lymphoma subtypes, we refer to excellent reviews [46,47,48,49].

### 4.1. Diffuse Large B-Cell Lymphoma

DLBCL constitutes ~35% of B-NHLs and is a morphologically, genetically and clinically heterogenous disease. DLBCL subtypes are commonly classified according to their cell of origin (COO), delineating GCB-DLBCL, ABC-DLBCL and an unclassified subtype [50,51]. The ABC-DLBCL subtype is associated with an unfavorable prognosis and shows a more aggressive disease course compared to its GCB-DLBCL and unclassified DLBCL counterparts. Recent genomic studies that molecularly profiled (mRNA, DNA alterations) large numbers of DLBCL cases have provided a more refined subdivision of DLBCL cases into genetically defined subgroups [52,53,54], ranging from 4 [53] and 5 [54] to 39 [52] subtypes. Whilst the ultimate goal should be to classify all DLBCLs according to their molecular lesions in order to personalize DLBCL treatment, currently the COO classification is still useful; for example, only 50% of cases fall into the classification scheme by Schmitz and colleagues [53], with the remaining cases being attributed to ABC-, GCB- or unclassified DLBCL subtypes.

More than 80% of ABC-DLBCL cases show genetic aberrations leading to aberrant NF-κB activation, which is considered a hallmark of this DLBCL subtype [6]. Initially, genetic mutations have been identified in regulators of the BCR (*CARD11*, *CD79A*, *CD79B*) and Toll-like receptor (*MYD88*) signaling pathways, leading to dysregulated NF-κB activation downstream of these pathways, as well as in a negative regulator of the NF-κB pathway (*TNFAIP3*, encoding A20) [55,56,57,58,59,60,61]. Mutations have subsequently been identified in several other NF-κB pathway components that lead to constitutive NF-κB activity in ABC-DLBCL [57,62,63]. Recently, an oncogenic BCR signaling supercomplex (MyD88-TLR9-BCR) has been identified in ABC-DLBCL which has been shown to activate NF-κB [64]. This complex facilitates synergistic signaling and crosstalk by clustering the BCR, CD79a, CD79b, MyD88 and associated proteins, including the IKKs, on the endolysosome membrane to allow for efficient downstream signaling largely through NF-κB. Tumors with the MyD88-TLR9-BCR supercomplex were generally associated with sensitivity to inhibition of the BCR signaling pathway by ibrutinib (see below) [64].

Only a small fraction of GCB-DLBCLs show NF-κB activation [6,57]. Amplification of the *REL* locus (encoding c-REL) can be detected in a subset of GCB-DLBCL cases; however, *REL* amplification is not associated with nuclear c-REL translocation [65]. It has therefore been proposed that enhanced c-REL activity provided by the amplification may have been required early in GCB-DLBCL pathogenesis, before becoming dispensable for tumor growth at later stages of tumor development [66].

Among the recently identified molecular DLBCL subtypes, genetic aberrations in NF-κB pathway components occurred predominantly in an ABC-DLBCL-related subset with MYD88^L625P^ and CD79B aberrations (MCD [53]; cluster 5 [54]) and in a subset with *BCL6* fusions, *NOTCH2* mutations and *TNFAIP3* lesions (BN2 [53]; cluster 1 [54]) that includes cases of ABC-, GCB- and unclassified DLBCL. Regardless of the DLBCL classification method, the pattern indicates that genetic aberrations in NF-κB pathway components in DLBCL predominantly correlate with a more aggressive clinical course. This notion is supported by the finding of Reddy and colleagues that genetic mutations in *NFKBIA* (encoding IκBα) occur in the GCB-DLBCL subtype, which is usually considered to show favorable prognosis, and are associated with a poor prognosis [52].

In DLBCL, the vast majority of alterations in NF-κB pathway components activate the canonical pathway. Mutations leading to aberrant activity of the alternative pathway were observed in ~10% of DLBCL cases and can occur in both ABC-DLBCL and GCB-DLBCL subtypes [67]. Of note, altogether ~25% of cases show nuclear p52 translocation [57], suggesting a broader role for this pathway in DLBCL pathogenesis. Mutations frequently target *TRAF2* and *TRAF3* that normally control the degradation of the central upstream regulator NF-κB-induced kinase (NIK) [67], thus constraining the alternative pathway from being activated. The role of aberrant NIK activity in driving DLBCL lymphomagenesis has been verified in a mouse model with constitutive transgenic NIK and BCL6 expression in GC and post-GC B cells [67].

The observation that the canonical or alternative NF-κB pathways can be found activated by genetic mutations in DLBCL may reflect the heterogeneity with regards to the cellular counterparts of the lymphomas. In normal GC B cells, both the canonical subunit c-REL, and in a mutually exclusive fashion, the alternative NF-κB subunits were found to be required for the maintenance of the GC reaction (see above). Aberrant activity of the subunits may promote tumorigenesis in the DLBCL-precursor cell, which could be an antigen-selected light zone B cell that is instructed to recirculate to the dark zone, by disturbing the normal dynamics of this developmental step [68]. ABC-DLBCL is thought to originate from a later developmental stage in the GC, i.e. from a selected GC centrocyte that is destined to differentiate into a PC. It will be interesting to determine whether RELA is the major canonical NF-κB subunit involved in the pathogenesis of ABC-DLBCL with aberrant NF-κB acticity, as this subunit was found to be required for PC development in vivo (Figure 2). Dysregulated RELA activity may promote lymphomagenesis by forcing a biological program onto the cell that is related to aspects of PC physiology.

### 4.2. Mantle Cell Lymphoma

MCL is a rare B-NHL subtype that is characterized by an aggressive clinical course [69]. The normal cellular counterpart of conventional MCL, the most common form of MCL, is thought to be an activated mature B cell with no or few of IgV hypermutations, indicating a pre-GC origin [70] (Figure 3). Although the fraction of MCL cases showing aberrant NF-κB activation and/or genetic mutations in NF-κB pathway components is presently unknown, it seems that similar to DLBCL, MCL cases can have either the canonical or alternative NF-κB pathway activated [69]. MCL cell lines that are sensitive to inhibitors of BCR signaling showed activation of the canonical NF-κB pathway [71]. Of note, cell lines insensitive to BCR inhibition displayed activation of the alternative pathway [71]. In accordance, recurrent genetic aberrations in the upstream regulators *TRAF2* and *BIRC3* were identified in ~15% of patients [71]. Interestingly, MCL lines with alternative NF-κB-pathway activity were sensitive to the pharmalogical inhibition of IKKβ, a central activator of the canonical pathway [71]. This finding suggests crosstalk between the two NF-κB pathways in MCL, at least in these cell lines. If the MCL precursor cell indeed represented an activated pre-GC B cell, one may speculate that, based on the in vivo observation that c-REL is required for B-cell activation prior to the GC reaction, whereas RELA is dispensable at this step [32] (Figure 2), c-REL may represent the critical NF-κB subunit in MCL pathogenesis (Figure 3).

### 4.3. Multiple Myeloma

MM is an aggressive cancer of PCs. Molecularly heterogeneous genetic aberrations cause dysregulated expression of various proto-oncogenes or inactivation of tumor suppressor genes, thus promoting MM pathogenesis [45,72]. Both the canonical and alternative NF-κB pathways can be activated in MM by signals from the bone marrow microenvironment. Nuclear translocations of RELA and RELB were observed in around 80% and 40% of primary MM cases, respectively [73,74]. Several groups have identified genetic mutations in NF-κB pathway components causing constitutive NF-κB activity in ~20% of primary MM and ~40% of MM cell lines [73,75,76,77,78,79,80]. The fraction of NF-κB-mutated cases may be higher, as an NCRI Myeloma-XI trial-based copy-number alteration study revealed that half of cases carried at least one deletion of NF-κB-pathway genes [81].

The genetic aberrations in MM are thought to allow for stromal-independent tumor-cell growth. ~70% of the mutations target components of the alternative NF-κB pathway, including amplifications and translocations of *MAP3K14* (encoding NIK), *CD40* and *LTBR* (encoding lymphotoxin-β receptor), and deletions or inactivating mutations of *TRAF3/TRAF2*, and *cIAP1/2* (*BIRC2/3*). All of these alterations lead to aberrant expression of NIK, the major usptream regulator of the alternative pathway. ~30% of the mutations are associated with activation of the canonical NF-κB pathway. Besides *CD40*, which activates both canonical and alternative NF-κB signaling, these include *CYLD*, *TACI*, and *NFKB1* (encoding p105/p50). Somewhat surprisingly, MM cell lines with aberrant NIK expression also showed activation of the canonical pathway [73,75,76,82]; shRNA-mediated knockdown of NIK and the pharmacological inhibition on IKKβ, the central regulator of the canonical pathway, was toxic to these cell lines. This indicates that NIK-mediated activation of the canonical NF-κB pathway was required for MM cell line growth [73,83,84]. Since shRNA-mediated knockdown of the alternative NF-κB subunits RELB or p52 in MM lines, as expected, was reported to cause cell death [74,85], the findings by Annunziata et al. suggest that both NF-κB pathways are active and promote cell growth/survival in MM in cases with genetic mutations, leading to constitutive NIK expression (which comprise a major fraction of NF-κB-mutated MM cases). Future work will determine to what extent the inhibition of either NF-κB pathway (or their downstream subunits) is sufficient to interfere with MM tumor cell growth, or whether both pathways need to be functionally ablated, as proposed by others. Of note, since the canonical subunit c-REL does not appear to be expressed in PCs and MM [31,43], the development of therapeutic strategies to inhibit the canonical pathway may focus on RELA/p50.

### 4.4. Other B-Cell Malignancies

Aberrant activation of NF-κB signaling occurs in several indolent lymphomas (Table 1). CLL is a non-Hodgkin lymphoma of the elderly where activation of the NF-κB pathway is long known to be involved in the pathogenesis [86]. Importantly, NF-κB is activated by the tumor microenvironment [87], upon which signals are propagated intracellularly, following signaling through NF-κB-activating cell surface receptors, including BCR, TLR, CD40 and BAFFR. Furthermore, epigenetic modifications and aberrant methylation of the genes-encoding tumor-suppressing miRNAs have been shown to activate NF-κB in CLL [88]. Although a wide range of genetic mutations in both canonical and alternative pathway components have been identified in CLL (including *MYD88*, *NFKBIE* and *BIRC3*) [89,90,91,92,93,94,95], these mutations occur at a low frequency [95]. Proceeding on the finding that the canonical subunit RELA was implicated in fludarabin resistance [96], it will be interesting to dissect the functions of the individual NF-κB pathways and subunits in CLL pathogenesis.

The finding that more than 90% of cases of Waldenström Macroglobulinemia (WM), a non-Hodgkin lymphoma with plasmacytic features, have *MYD88*^L265P^ mutations [97,98] implicates a crucial role of the aberrant activity of the canonical NF-κB pathway in this disease.

Constitutive NF-κB-pathway activation is a hallmark of Hodgkin lymphoma (HL) [99,100]. A large fraction of HL cases are associated with Epstein-Barr virus (EBV), the genome of which encodes LMP1, a potent NF-κB activator. Genetic mutations in HL predominantly affect canonical NF-κB pathway components [101,102], with a subset of cases harboring aberrations in components of the alternative pathway [103,104]. Similar to GCB-DLBCL [51], HL and mediastinal large B-cell lymphoma (MLBCL; a DLBCL subtype that is phenotypically related to HL) show amplifications of the *REL* locus [105,106,107]. Unlike GCB-DLBCL [65], however, both HL and MLBCL cases display nuclear translocation of c-REL [105,108,109], implicating a role for c-REL in the maintenance of HL and MLBCL tumor cells. Also the alternative pathway is activated in HL cells, as signaling through CD30, CD40 and LMP1 activates both the canonical and alternative NF-κB pathways [99]. This is reflected by siRNA knockdown experiments of RELA-p50 and RELB-p52 in an HL cell line that showed a greater decrease in cell viability in the latter knockdown (95% vs. 40%) [110]. Of note, the effect of c-REL knockdown has not been tested in this study. It appears that in HL, the aberrant activity of both NF-κB pathways is involved in the disease mechanism. The same appears to be the case for splenic marginal zone lymphomas and mucosal-associated lymphoid tissue (MALT) lymphomas [47,48] (Table 1).

Finally, although in the human, Burkitt lymphoma is not normally associated with aberrant NF-κB activation, a mouse model that develops a MYC-driven, Burkitt-like disease represents a useful tool to study the mechanism of NF-κB-pathway components in tumorigenesis. Using the Eμ-Myc mouse model, Hunter and colleagues could attribute a pathogenic role to a single NF-κB subunit, namely c-REL [111], providing further rationale for the need to dissect the functions of the individual NF-κB transcription factor subunits in lymphoid malignancy.

## 5. NF-κB as a Therapeutic Target in Lymphoid Malignancies

The crucial role of aberrant activation of the NF-κB pathway in the pathogenesis of lymphoid malignancies have identified targeting of aberrant NF-κB signaling as a logical treatment strategy for these diseases [71,99,112]. Genetic aberrations upstream of Bruton tyrosine kinase (BTK) in the BCR signaling pathway can be successfully treated by a small molecule inhibitor of BTK, ibrutinib [113]. Besides the phosphoinositide 3-kinase (PI3K) pathway, BTK inhibition blocks the activation of the canonical NF-κB pathway. However, mutations in the BTK protein have been found in CLL and MCL, which confer resistance and limit the efficacy of the drug [114], indicating the need for alternative treatment strategies. Proteasome inhibitors prevent degradation of the canonical NF-κB inhibitor IκBα among many other proteins, in addition to the initiation of the unfolded protein response (UPR) by the build-up of protein, which is thought to contribute to their toxicity [115]. The first generation proteasome-inhibitor bortezomib was found to be highly effective in the treatment of refractory MM [116]. However, in MM cells, bortezomib was observed to induce canonical NF-κB activation [117,118], suggesting that bortezomib-induced toxicity in MM cells cannot be fully attributed to inhibition of canonical NF-κB activity. Similarly, it is not clear to what extent NF-κB inhibition contributes to bortezomib-mediated cell toxicity in MCL [119,120,121]. Nevertheless, bortezomib is currently used in combinatorial frontline therapies for MM and MCL [122]. In DLBCL, bortezomib showed initial success in the treatment of ABC-DLBCL, but not GCB-DLBCL, when combined with chemotherapy [123,124]. This may be explained by the activation of NF-κB through chemotherapeutic agents, although it was unclear whether the improved response in the combination treatment was due to bortezomib-induced NF-κB inhibition [123]. Small-molecule inhibitors of IKKβ activity are potent inhibitors of canonical NF-κB signaling in many in vitro settings [125,126]. However, although IKKβ inhibitors have been available since long and were tested in clinical trials, it appears their toxicity renders them unsuitable for therapeutic use [127,128,129,130]. Moreover, strategies to inhibit upstream NF-κB-pathway components need to take into consideration possible NF-κB-independent functions [131], as shown for IKKβ [128,132].

Overall, it emerges that current therapeutic approaches aimed at inhibiting aberrant NF-κB signaling lack specificity. More generally, a serious problem associated with the use of global NF-κB inhibitors in cancer therapy is that NF-κB activation virtually plays a role in every cell type. Therefore, treatment with NF-κB inhibitors could lead to systemic toxicity [133], thereby limiting their usability and efficacy.

In order to develop more effective therapies aimed at aberrant NF-κB activity while avoiding systemic toxicity, strategies are required to selectively target the specific NF-κB signaling components that are oncogenic within the malignancies. It is becoming clear that understanding the mechanism of pathogenic NF-κB activity in malignancy must focus on identifying the oncogenic functions of the separate NF-κB transcription factor subunits—the downstream mediators of NF-κB activation [134], and of the specific target genes of these subunits [122]. Initial efforts in this direction have been made. A promising approach is to target the survival-mediating, downstream effectors of NF-κB, as recently described for the GADD45β/MKK7-complex in an MM xenograft model [135]. Additionally, a newly developed small molecule c-REL inhibitor showed anti-cancer cell properties in a xenograft model [136], although this inhibitor may not be c-REL-specific as it inhibited RELA function in another study [137].

Proteolysis-targeting chimeras (PROTACs) are a novel class of compounds that harness the power of the ubiquitin–proteasome pathway to selectively degrade cellular proteins of interest, thus comprising a specific targeting strategy. The term was coined in 2001 following the generation of a compound which recruited a protein of interest to an E3-ligase via two binding moieties attached by a linker [138]. Proof of principle work using a PROTAC to degrade BRD4 in lymphoid malignancies was independently shown in Burkitt lymphoma [139], acute myeloid leukemia [140], and MCL [141]. More recently, BTK was successfully targeted by PROTAC-mediated degradation in ibrutinib-sensitive and resistant lymphoma cell lines, which may comprise a promising strategy for the treatment of BTK-resistant tumor cases [142]. In this study, the corresponding PROTAC demonstrated a reduction in off-target effects on other kinases normally implicated by the use of ibrutinib [142]. Overall, the work on PROTAC strategies thus far demonstrates the scope of this approach for the specific targeting of intracellular proteins and could hence be harnessed to specifically target downstream NF-κB subunits or their effectors.

## 6. Conclusions and Outlook

There is increasing evidence that in certain lymphoid malignancies, the oncogenic effects of aberrant activation of the NF-κB signaling pathway, either via the tumor microenvironment or through cell-intrinsic genetic mutations, can be mediated by distinct downstream effectors. These effectors include the canonical and alternative NF-κB pathways, and the separate NF-κB transcription factors as well as their target genes. This selectivity may be exploited for the development of more specific NF-κB-inhibitory therapies. In tumor entities where the activity of both the canonical and alternative NF-κB pathways has been implicated in the pathogenesis (e.g., HL, MM) (Table 1), it will be important to identify the individual contributions of either pathway, and to determine the extent to which the functional ablation of the separate pathways affects tumor cell growth or survival. These efforts are expected to provide the rationale and mechanistic basis for the development of more specific, less toxic NF-κB inhibitors against these malignancies.

It is becoming increasingly clear that the separate NF-κB subunits have known pathogenic roles in many cancers, either within the cancer cell or in cells of the tumor microenvironment [143,144,145]. This includes inflammation-associated cancers, which has implications for immunotherapy. As a recent example, specifically ablating c-REL function in murine Treg cells delayed melanoma growth by impairing Treg-mediated immunosuppression, and potentiated the effects of anti-PD-1 immunotherapy [146]. Therefore, understanding the roles of individual NF-κB subunits in both tumor cells and cells of the tumor microenvironment has the potential to inform the development of new treatment strategies.

## Figures and Tables

**Table 1:** Characteristics of lymphoma subtypes and NF-κB involvement.

Subtype			Characteristics & Main Genetic Aberrations		NF-κB Involvement
Non-Hodgkin’s Lymphoma	BL		Aggressive t(8;14) *MYC*		Rare or no involvement of NF-κB [147].
	CLL		Indolent Highly heterogeneous disease, comprising two main subtypes 60% are *IGHV*-mutated (good prognosis) and 40% are *IGHV*-unmutated (poor prognosis) [86,148]. Large range of genetic aberrations [95].		NF-κB pathway constitutively active by stimulation of cell surface receptors and tumor–microenvironment interactions. Genetic mutations in NF-κB pathway components affect both the canonical and alternative pathway, but occur at low frequency (See text).
	DLBCL	ABC-DLBCL	Genetic mutations in genes encoding histone/chromatin modifiers; genetic abnormalities targeting *BCL6*; evasion of immune surveillance by lack of MHC class I expression [6,149].	Aggressive *MYD88; CD79A/B; TNFAIP3; PRDM1; CDKN2A/B; CARD11*	Constitutive NF-κB pathway activation in >80% of cases. Genetic mutations in various NF-κB pathway components, including *CARD11, CD79A/B, MYD88, TNFAIP3, TRAF3*. Only 10% of mutations affect the alternative pathway, although 25% of cases show nuclear p52, includes MCD [53] and cluster 5 [54] subtypes, as well as a fraction of BN2 [53] and cluster 1 [54] subtypes (See text).
		GC-DLBCL		Indolent * *BCL2; MYC* translocations; *EZH2; GNA13; PTEN*	Constitutive NF-κB activation is rare. *REL* amplification, although lack of correlation with nuclear c-REL translocation. Includes fraction of BN2 [53] and cluster 1 [54] subtypes and the aggressive subtype with genetic mutations in *NFKBIA* [52] (See text).
		Unclassified	Indolent Lymph-node signature [51].		Includes fraction of BN2 [53] and cluster 1 [54] subtypes (See text).
		PMBCL	Aggressive Affects young adults (predominantly females).		Constitutive NF-κB signaling. *TNFAIP3* is mutated in ~35% of cases [101]. *REL* amplification in 20–50% of cases (See text.)
	FL		Indolent (aggressive following histological transformation to DLBCL) [150,151] t(14;18) *BCL2*		NF-κB involvement in transformed FL [152,153].
	MCL		Aggressive t(11;14) *CCND1* Additional diverse molecular events: deregulation of cell cycle, DNA damage repair, apoptosis and chromatin modifiers [69].		Canonical NF-κB pathway activation (in cell lines sensitive to BCR inhibitors) and alternative pathway activation (in cell lines insensitive to BCR inhibitors). Recurrent mutations in *TRAF2* or *BIRC3* in 15% of patients (See text).
	MZL	MALT	Indolent t(11;18) *c-IAP2* and *MALT1* fusion [47,48] *Helicobacter Pylori* infection associated [154]		*c-IAP2* and *MALT1* fusion protein induces robust activation of canonical and alternative NF-κB pathways. *TNFAIP3* deletions [47,48].
		Other	Indolent		*TNFAIP3*. [155] Activate canonical and alternative pathway activation at almost equal fractions [156].
	WM		Indolent IgM-secreting lymphoplasmacytic lymphoma *MYD88* [97,98]		Gain of function *MYD88* mutation in 90% of cases, which activates the canonical pathway via TLR signaling (See text).
Hodgkin’s Lymphoma			Indolent ~50% EBV-associated [157]		EBV-associated LMP1 is a potent NF-κB activator. 20% and 40% inactivation/deletion of *NFKBIA* and *TNFAIP3* (preferentially in EBV-negative cases) *REL* amplification in ~40% of cases (See text.)
MM			Aggressive Molecularly heterogeneous genetic aberrations, including cyclin D/retinoblastoma pathway [45,72]		Constitutive canonical and alternative NF-κB pathway activation. NF-κB activating signals from tumor microenvironment. Majority of genetic alterations in NF-κB affect alternative pathway (>70%), including *MAP3K14* (NIK), *CD40*, *LTBR*, *TRAF2/TRAF3*, *cIAP1/2*, leading to aberrant NIK activity. Genetic mutations in canonical pathway include *CYLD*, *TACI*, and *NFKB1*. Aberrant NIK activation also activated canonical pathway (See text).

* aggressive GC-DLBCL has also been described: The ‘double-hit lymphomas’ (DHL) are characterized by translocation of *MYC and BCL2* (or less commonly *BCL6*) [149]. ABC, activated B cell; BL, Burkitt lymphoma; CLL, chronic lymphocytic leukaemia; DLBCL, diffuse large B-cell lymphoma; EBV, Epstein-Barr virus; FL, follicular lymphoma; GC, germinal center; HL, Hodgkin’s lymphoma; MCL, mantle cell lymphoma; MALT, mucosa-associated lymphoid tissue; MM, multiple myeloma; MZL, marginal zone lymphoma; PMBCL, primary mediastinal B-cell lymphoma; WM, Waldenström’s macroglobulinemia.
